# 5G V2X Performance Comparison for Different Channel Coding Schemes and Propagation Models

**DOI:** 10.3390/s23052436

**Published:** 2023-02-22

**Authors:** Dimitrios Chatzoulis, Costas Chaikalis, Dimitrios Kosmanos, Kostas E. Anagnostou, George T. Karetsos

**Affiliations:** 1 Department of Digital Systems, School of Technology, University of Thessaly, Geopolis Campus, 41500 Larissa, Greece; 2 Department of Electrical & Computer Engineering, University of Thessaly, 38221 Volos, Greece; 3 Department of Informatics & Telecommunications, University of Thessaly, 35100 Lamia, Greece

**Keywords:** 5G-NR, 4G-LTE, V2X, turbo codes, LDPC, polar codes, stochastic V2X model, implementation scenarios

## Abstract

Channel coding is a fundamental procedure in wireless telecommunication systems and has a strong impact on the data transmission quality. This effect becomes more important when the transmission must be characterised by low latency and low bit error rate, as in the case of vehicle-to-everything (V2X) services. Thus, V2X services must use powerful and efficient coding schemes. In this paper, we thoroughly examine the performance of the most important channel coding schemes in V2X services. More specifically, the impact of use of 4th-Generation Long-Term Evolution (4G-LTE) turbo codes, 5th-Generation New Radio (5G-NR) polar codes and low-density parity-check codes (LDPC) in V2X communication systems is researched. For this purpose, we employ stochastic propagation models that simulate the cases of line of sight (LOS), non-line of sight (NLOS) and line of sight with vehicle blockage (NLOSv) communication. Different communication scenarios are investigated in urban and highway environments using the 3rd-Generation Partnership Project (3GPP) parameters for the stochastic models. Based on these propagation models, we investigate the performance of the communication channels in terms of bit error rate (BER) and frame error rate (FER) performance for different levels of signal to noise ratio (SNR) for all the aforementioned coding schemes and three small V2X-compatible data frames. Our analysis shows that turbo-based coding schemes have superior BER and FER performance than 5G coding schemes for the vast majority of the considered simulation scenarios. This fact, combined with the low-complexity requirements of turbo schemes for small data frames, makes them more suitable for small-frame 5G V2X services.

## 1. Introduction and Literature Review

Intelligent Transportation Systems (ITSs) represent efficient solutions for improving traffic safety and efficiency, as well as providing infotainment services. V2X communications include vehicle-to-vehicle (V2V), vehicle-to-network (V2N) or vehicle-to-infrastructure (V2I), vehicle-to-road side unit (V2R) and vehicle-to-pedestrian (V2P) communications, and they represent the key enablers of ITSs [1]. V2X communication helps vehicles to obtain and share information periodically (also known as beaconing), such as location, speed, braking action, road and intersection status, to improve safety. ITSs were introduced in 1999 by the US Federal Communication Commission, and they immediately triggered significant research activity all over the world and especially in the USA [2] and Europe [3]. The research resulted in a first set of radio standards for V2X in the USA [4] and in Europe [5], respectively.

The first standard to present direct device-to-device (D2D) communications was 3GPP Release 12, which was originally developed for safety services [6]. 3GPP Release 12 introduced a new service function, the Proximity Service, which allows the devices to discover peer devices for D2D communication services and to directly communicate and exchange data with neighbouring entities. This initiative served as the base for the development of LTE V2X based on the 4G LTE under Release 14 [7] and 15 [8]. 3GPP Release 15 included 5G NR also, but it did not include sidelink aspects. Release 16 is the first to introduce V2X communications, including sidelink communications, based on the 5G NR air interface [9]. It focuses on enhancing V2X reliability, latency, capacity and flexibility and it also targets advanced use cases, such as platooning, extended sensors, advanced driving and remote driving [10]. All the above communication standards use the Forward Error Correction technique for controlling errors in data transmission over noisy communication channels.

On the other hand, turbo codes, based on the turbo principle [11], represented a revolutionary channel coding signal processing technique, introduced in 1993 [12,13], to achieve near Shannon performance [14]. The critical and most complex process is turbo decoding. Mainly, two decoding algorithms are used for turbo decoding. The MAP algorithm of Bahl, Cocke, Jelinek and Raviv (BCJR) [15], and the Soft-Output Viterbi Algorithm (SOVA) [16], which though simpler, has some deprivation in performance. BCJR is a complex algorithm, so the less complex but suboptimal Max-Log-MAP implementation of the algorithm [17,18] was proposed. Further, the Log-MAP implementation of the algorithm [19] was proposed, giving a performance close to that of the MAP algorithm and correcting the approximation used in the Max-Log-MAP. Turbo codes had superior performance, and due to their excellent error-correcting capability, they rapidly became part of 4G-LTE [20,21], but latency and complexity were drawbacks, mainly due to large frame sizes [22]. LDPC [23,24,25] is much simpler [26] than turbo coding. According to 3GPP TS 38.212 [27], LDPC is recommended for 5G NR shared channels due to its high throughput, low latency, low decoding complexity and rate compatibility [28,29]. On the contrary, it does not perform very well for a short code length and low code rate [30]. Polar codes [31] are the first provably capacity-achieving family of codes with low encoding and decoding complexity. Due to the good performance [28,29], polar codes are now being used to transmit control information in the 3GPP 5G-NR [32].

In [33], the authors showed that turbo codes can be used for enhancing safety-related 5G V2V and V2I services for short frame lengths and correlated (flat) Rayleigh fading channel. In [34] and [35], the authors investigated the impact of turbo codes on the quality of service and BER performance, considering a 3GPP 5G NR V2X environment and small length frames. In [36], several waveform candidates and coding schemes under a common framework were investigated for V2X use cases, requiring ultra-reliable and low-latency communications under harsh channel conditions. Dey et al. [37] proposed a model featuring an LDPC code that can improve reliability by reducing the bit error rate compared to the existing physical layer framework. In [38], they investigated the impact of LDPC and multiple input multiple output (MIMO) in a V2X environment, supported by Dedicated Short-Range Communication.

The performance of different coding schemes attracted the research community in various communication systems. In [39], the authors evaluated different channel coding techniques potentially applicable in IEEE 802.11be. Vaz et al. [40] focused on the BER evaluation and analysis of turbo and polar codes over AWGN channels for varying block lengths and code rates and, Belhadj et al. [41] analysed the impact of turbo, polar and LDPC codes for 5G massive-machine-type communications over the AWGN channel. In [42], the authors compared the 5G channel coding techniques, e.g., polar and LDPC for the AWGN channel. Thorough research has been conducted in Worldwide Interoperability for Microwave Access (WiMAX) as well. In [43], the performance of the WiMAX system using LDPC, turbo coding, MIMO and Rayleigh channel was simulated and analysed.

Based on the discussion above, a lot of work has been carried out in path loss analysis and modelling for V2V communications as well as the channel coding/decoding schemes in various wireless channel conditions. Moreover, V2X use cases require ultra-reliable and low-latency communications (URLLCs) under harsh channel conditions. To select a coding scheme for such use cases represents a key challenge. For this reason, in the published literature, no one has addressed the V2X channel modelling and coding issues combined in realistic road environments with the aim of designing a future adaptive model that will dynamically select the appropriate encoding/decoding scheme with the corresponding parameters providing a high quality of service (QoS) in driving experience. Therefore, in this work, we investigate and compare the performance of turbo codes, LDPC and polar codes for V2V traffic information for small frames in 3GPP 5G V2X Release 16 practical scenarios. Bearing in mind these V2V scenarios, we investigate which can be implemented and are more efficient. The idea is to use the derived experimental results in a future system of dynamic data rate selection based on the conditions of the external environment (e.g., traffic density, wireless interference, safety level) to achieve the optimal BER among all the possible coding schemes and combine them in a reconfigurable and adaptive coding scheme. The main contributions of this paper are:

- Simulation scenarios are investigated and are based on release 16 of the 3GPP 5G V2X technical report, incorporating the use of both turbo-based and 5G-NR coding schemes. Aiming at an extensive analysis of the behaviour of coding schemes, different traffic propagation models, channel states and frame sizes are considered.
- Analysis of the simulation results focusing on comparing the BER performance versus the SNR for the BCJR, logBCJR, maxlogBCJR, SOVA, LDPC and polar decoding algorithms in the implemented scenarios. The main objective is to document BER efficiency in small data frames and extract the optimal decoding scheme in the case of small-frame 5G V2X environments.
- The simulation results are used to study the impact of QoS parameters on the system performance and on the decision of the optimal decoding scheme. Finally, the additional knowledge resulting from this work can be used to build a coding scheme, exploiting machine learning (ML) techniques, where the ML coding scheme will learn using data from the best coding/decoding technique for a given SNR and frame size. This scheme will consider not only BER performance but also QoS parameters aiming for optimal performance in dynamic 5G V2X communication environments.

## 2. Proposed V2X Communication System Model

### 2.1. 3GPP 4G-LTE and 5G-NR Traffic Scenarios and Channel States for V2X Communication Models

A V2X communication system model simulates the data transmission between two moving entities. Two main traffic scenarios are considered both for 4G-LTE [7] and 5G [8]. In the first scenario, called urban, the vehicles are moving in an urban area that may have many obstacles and scatterers. In the second scenario, called highway, the vehicles are moving in a freeway–highway area, where the obstacles and scatterers affect the propagation path less. According to 3GPP [7,8], a V2X communication channel link between two vehicles can have three different states:

- LOS: A V2V link is in LOS state if the two vehicles are in the same street and the propagation path is not blocked by other vehicles nor other objects.
- NLOSv: A V2V link is in NLOSv state if the two vehicles are in the same street and the propagation path is blocked by other vehicles.
- NLOS: A V2V link is in NLOS state if the two vehicles are in different streets and the propagation path is blocked by blockages such as buildings. Obviously, the NLOS state cannot be implemented in the highway scenario, as stated in [7], because the vehicles are always moving in the same street.

### 2.2. Large-Scale Fading in V2X System Modelling

Large-scale fading is a function of two main factors, path loss and shadowing [44,45,46]. Path loss models the average power attenuation in V2X channels, is deterministic and it is a function of the three-dimensional (3D) distance d between the two vehicles in meters and the carrier frequency $f_{c}$ in GHz. The determination of large-scale modelling for V2X has been of great concern. In [47], they studied the vehicle-induced path loss for millimetre-wave V2V communication. M. Yang et al. [48] focused on the analysis and modelling of path loss characteristics for V2V communications with vehicle obstructions. In [49], the authors investigated the performance parameters of path loss and shadowing in different 5G NR V2V communication environments. Further, in [50], the authors validated the channel model that 3GPP proposed for NR-V2X systems and highlighted potential density-based inconsistencies in the model and provided recommendations for future measurement campaigns in vehicular environments. Traffic density, the 3D shape of the vehicles, i.e., length, width and height, and the position of the antennas were taken into account [51] to estimate the path loss in V2V communications and to predict the SNR distribution and the service probability. Boban et al. [52] obtained state transition probabilities for LOS, NLOS and NLOSv channel states that can be used to generate time-evolved V2V channel path loss realisations for representative urban and highway environments. Further, in [53], the authors designed a geometry-based efficient propagation model for V2V communication called GEMV^2^ to simulate large-scale signal variations deterministically for city-wide networks based on geometric characteristics of the environment. In [54], the authors analysed and modelled large-scale fading by characterising the influence of common shadowing objects, both regarding the autocorrelation and the joint multilink cross-correlation process for communication at 5.9 GHz. The basis of the aforementioned research lies in the 3GPP technical reports for 4G-LTE and 5G-NR V2X [7,8] communications. Accordingly, the path loss in dB is computed by deterministic functions that are summarised in Table 1.

The second factor, i.e., shadowing, is stochastic and it is implemented as a log-normal random variable that models the variations in power loss due to scattering that exist in the communication system. The shadow fading models for all types of 5G-NR V2X systems are described in [8] and they are adopted from [7] for LTE V2X. Table 2 presents the shadow fading parameters for each possible state and model.

When a V2X link is in NLOSv state an additional blockage loss in dB is summed to the path loss, this loss models the possibility that moving vehicles may block the signal between the transmitter and the receiver. This blockage loss a is modeled according to a log-normal random variable, with mean $\mu_{a}$ and standard deviation $\sigma_{a}$, $\alpha ∼Lognormal\left(\mu_{\alpha },\sigma_{\alpha }^{2}\right)$. Table 3 presents the parameters of the NLOSv additional blockage. In summary, the total path loss of a V2X link is summed up in Table 4.

### 2.3. Small-Scale Fading in V2X System Modelling

Extensive research has been conducted lately in order to determine small-scale fading parameters. Fan et al. [55] used the Akaike Information Criterion, which aims to solve the problem of the optimal model selection and to select the best fading distribution for vehicle radio channel between Rayleigh and Rician distributions in a highway environment. They showed that the Rician distribution has the best fitting for the V2V radio channel in a highway communication environment and found that the vehicles, as obstacles have the largest influence on the small-scale fading of V2V channels by modelling the time-varying nature of the K factor. In [56], a small-scale fading analysis of the V2V channel inside tunnels was presented, driven from narrowband channel measurements, and it was collected in four different tunnels under real road traffic conditions. The Rice distribution has been proven to provide a reasonable fit to the measured small-scale fading, whereas the measured time-varying K-factor estimation inside tunnels is between 3 and 6.4 dB and outside tunnels is between 4.9 and 8.6 dB.

In [57], K-factor estimation in Urban Terrain for Gdansk city (base station to vehicle) was presented. The authors proposed a Gaussian mixture for K-factor distribution in urban terrain after measuring the data. In [58], the authors studied V2V channel characteristics at the 5.9 GHz band based on measurement under a ramp, with a soundproof wall on the urban viaduct and an urban intersection scenario. The authors concluded that for the NLOS case, a Weibull distribution more appropriately describes the propagation medium, and a Rician distribution is the best for describing the LOS case. In [59], data captured from typical urban, suburban and rural areas were analysed and the Rician K factor was approximated for each scenario to give an insight into the V2V channel behaviour. For all three areas, most K values were found to be within a range of −10 to +10 dB, concluding that the logistic distribution provided a somewhat better fit, with very similar mean and standard deviation.

The aforementioned results are mainly based on experimental measurements with real data or geometry-based simulations. 3GPP technical reports for 4G-LTE and 5G-NR V2X communications [7,8] provide a stochastic approach to small-scale fading. The channel state characterises the type και conditions of data transmission. LOS and NLOSv states are types of data transmission where there is a dominant LOS signal between the transmitter and the receiver, although in the second case, the dominant path is partially obscured by other vehicles. For this purpose, the channel in LOS and NLOSv cases is modelled as a Rician channel [7,8]. Due to the randomness and high mobility in the communication environment, both in 4G-LTE and 5G-NR V2X system modelling, the parameters of the Rician channel are also random. Therefore, the K factor of the Rician channel is determined as random following a log-normal distribution in dB. The mean and standard deviation of the K factor are presented in Table 5. For the NLOS state, a Rayleigh channel is assumed to be more suitable.

In the V2V system model, the transmitter and the receiver are highly mobile, so a dual mobility model must be considered with different Doppler impacts. The model must consider the relative speed between the transmitter and the receiver. Specifically, the Doppler shift is expressed according to [8] (p. 31):

### 2.4. Simulation System Model

**Figure 1:**
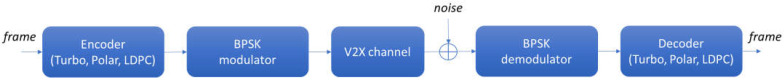
Simulation model using V2X stochastic channel model and BPSK modulation.

Figure 1 shows the simulation system model that is used throughout this article.

In our simulation model we implement and use six coding/decoding schemes: BCJR, logBCJR, maxlogBCJR and SOVA turbo coding, polar and LDPC coding. The process of turbo coding is implemented according to [20]. Although the implementation of the coding scheme is identical for every algorithm, the decoding procedure varies among BCJR [15], maxlogBCJR [17,18], logBCJR [19] and SOVA [16]. Turbo coding is implemented using four iterations, based on the recommendation given in [33] for V2X communications, which was confirmed by the simulations. Polar coding and decoding were implemented according to [60], where the authors briefly describe the implementation based on [32]. LDPC coding and decoding were implemented according to the offset min-sum algorithm, where the base matrices are provided in the NR standard specification TS 38.212 [32]. From our simulations, we concluded that after 12 iterations, the results reach an equilibrium, so we chose to use 12 iterations for each implementation of the LDPC decoder. Although polar and LDPC in 5G NR have flexible coding rates set, turbo coding has a fixed coding rate in 4G-LTE equal to 1/3. This is the reason that we chose a coding rate value of 1/3. Subsequently, the frame data are modulated at the transmitter and demodulated at the receiver by a Binary Phase-Shift Keying (BPSK) modulator and demodulator, respectively, as depicted in Figure 1.

The V2V mobile channel is implemented according to Section 2. Our goal is to implement a V2V model, where accuracy meets generality [61] in a simple and fast simulation framework, since our communication model is less complex compared to geometry-based models, without significant losses in accuracy. That is why we decided to implement a stochastic model and avoided using a geometry-based model, similarly to [53] and [62]. Therefore, for a realistic simulation setup, we use a stochastic V2V channel using the model described in [8]. A carrier frequency equal to 5.9 GHz is also considered, similarly to [34]. We also assume that the vehicles are moving at a constant speed of 50 Km/h in an urban environment and at a constant speed of 100 Km/h in the highway traffic model.

Large-scale fading is computed according to Table 4, where we assume that the distance between the antennas of the two vehicles is 5 m. Further, for the NLOSv state, we assume that each case (Table 3) is equiprobable. Small-scale fading is computed as flat Rayleigh for the NLOS case and flat Rician for the LOS and the NLOSv case, where the parameters of the channels are derived from Table 5 and calculated separately for each frame. Path modelling is implemented using a Jakes Doppler spectrum, with a Doppler shift that can be specified by [8] (p. 31). Jakes model is created according to the sum of the sinusoids technique, where eight sinusoids are used, similar to [33]. Moreover, in [63], a similar V2X channel model was used with 16 sinusoids. Thus, our channel model is verified to be used in V2X systems.

## 3. Simulation Scenarios

In our simulations, we assume three different short data frame sizes, consisting of 128, 256 and 512 bits, respectively, as explained in [34,35]. Furthermore, we implement 5 different simulation scenarios for each data frame size and, totally, 15 simulation scenarios, which are summarised in Table 6. For each simulation scenario, we transmit 100,000 frames from the transmitter to the receiver through the mobile channel. Furthermore, MATLAB simulation tool was used for the generation of the results using floating-point precision.

## 4. Simulation Results

Our main goal is to investigate the behaviour of the V2X communication channel in different conditions, for different coding algorithms and for different frame sizes. To this end, we focus on comparing the decoding algorithms for each scenario in Table 6 and, subsequently, we examine their effectiveness by investigating their BER performance in these scenarios.

### 4.1. Simulation Results for 128-Bit Frame Length

**Figure 2:**
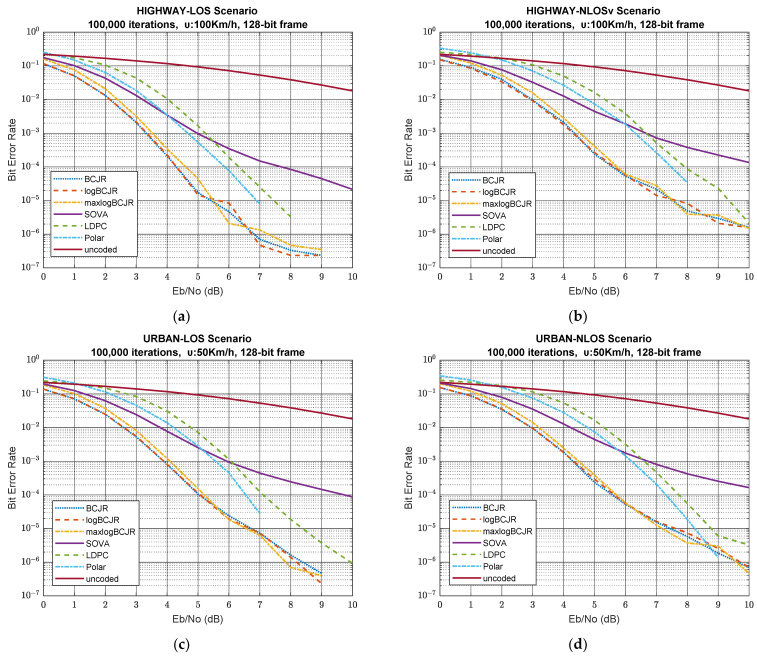

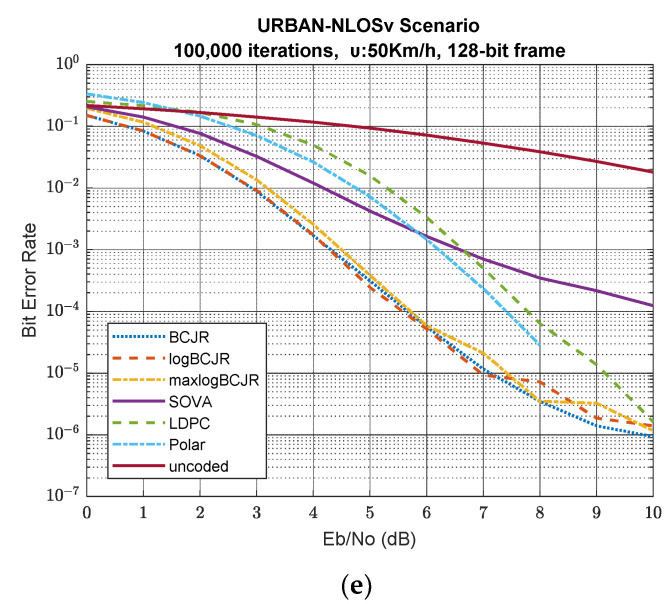
BER vs. SNR for scenarios 1-5 and frame length 128 bits. (**a**) Highway-LOS scenario; (**b**) highway-NLOSv scenario; (**c**) urban-LOS scenario; (**d**) urban-NLOS scenario; (**e**) urban-NLOSv scenario.

Figure 2 illustrates BER performance of the simulated system for a 128-bit frame, i.e., for scenarios 1-5 in Table 6. We observe that every decoding algorithm improves the BER as the SNR increases at a rate superior to the uncoded case. BCJR and logBCJR coding algorithms have identical behaviour. MaxlogBCJR has a loss of 0.2-0.3 dB at the same BER relative to BCJR and logBCJR in all scenarios, which is expected, since their performance is suboptimal [64].

Apart from gap, all three algorithms have the same rate of convergence, and above a threshold, an error floor appears [65], limiting the rate of BER convergence. This error floor is smooth for all five scenarios, and it always appears between 5 and 7 dB SNR. Despite the presence of the error floor, these three algorithms have a high rate of reduction. The SOVA decoding algorithm is also suboptimal [66] relative to the turbo-based algorithms. Further, it has better performance compared to LDPC and polar coding for low SNR. As the SNR increases, the BER decreases at a slow pace and, eventually, SOVA becomes the worst of all the coding algorithms above 5.5 to 6.8 dB according to the scenario.

Polar coding has the worst initial BER (0 dB), worse than the uncoded case, but as the SNR increases, BER diminishes rapidly in all scenarios. The result is that the BER nulls for SNR greater than 7 dB in highway-LOS and urban-LOS scenarios. Further, it nulls above 8 dB in highway-NLOSv and urban-NLOSv scenarios and above 9 dB in the urban-LOS scenario. LDPC has a much slower rate of convergence for low SNR, and as the SNR increases, the slope of the BER gradually increases in all 128-bit scenarios. This diminishing rate slightly increases in all scenarios except the highway-LOS scenario, indicating the presence of an error floor [67,68], which is also present in LDPC, limiting its performance.

Table 7 shows the SNR needed to achieve a BER of value 0.001 and 0.000001 for all the aforementioned scenarios and for the 128-bit frame length. If our goal is to achieve BER equal to 0.001, then BCJR-based algorithms need less signal power than the rest of the algorithms. If our goal is to achieve BER equal to 0.000001, instead, then polar is the best coding scheme, as it is the only algorithm that achieves this objective in all five scenarios.

Analysing the performance of the first two scenarios, we can extract a qualitative measure of the impact that the highway traffic model has on different channel states (LOS, NLOSv). The presence of moving obstacles in the NLOSv state has an impact on BER by approximately an order of magnitude, on average, as shown by Table 8.

The first and third scenario involve having LOS channel state and different traffic environments, urban and highway, respectively. If we compare them, we can observe the effect of the traffic model on the system performance. As we can deduce, the effect of the model is greater for small SNR and decreases as the SNR increases. Table 8 shows the achieved BER values for SNR equal to 4 and 9 dB, respectively. The highway traffic model creates 3- to 4-times smaller BER than the urban traffic model for SNR up to 4 dB, but as the SNR increases, the difference is significantly smaller between the two scenarios. The main cause of this phenomenon is that, as the SNR remains low, the multipath is scattered by the rich scatterers, uniformly distributed around the receiver dominating the LOS component. Nevertheless, as the SNR rises, the LOS signal component dominates among all other scattering components, making them negligible [69]. This means that as the SNR increases, the traffic model has less impact on BER performance.

Scenarios 3, 4 and 5 refer to the urban traffic model and different channel states (LOS, NLOS and NLOSv). Comparing these three scenarios, we can observe the effect of the channel state on system efficiency. From Table 8, we infer that the presence of a dominant LOS component has a great impact on BER performance, especially for algorithms that can converge fast at low SNR. BER for BCJR and logBCJR is, in the third scenario, 40-times smaller than the other two urban scenarios when the SNR equals 4 dB. Contrariwise, BER is 2- to 3-times smaller for the rest of the algorithms and for the same SNR, mainly because these algorithms are characterised by a slow rate of convergence at low SNR regions. When the SNR increases, this gap gradually narrows, although LOS channel state still has superior performance among the three scenarios.

If we focus our study on the last two scenarios, we can easily conclude that for low SNR, we obtain identical performance. As the SNR rises, noise has less impact on the communication channel and the signal is dominant. If we consider that the NLOSv state comes from three equiprobable transmission cases, we can assume that the NLOSv state will have slightly better performance compared to the NLOS state. This fact can be seen from Table 8, where BER is slightly better in the fourth scenario than in the third one.

Scenarios 2 and 5 are based on NLOSv channel state. Although they have different traffic environments, they have common small-scale features, except for the Doppler effect. BER for these two scenarios is identical when the SNR is low, and as the SNR increases, the second scenario has a little better performance. Bearing in mind that the NLOSv state is a partially bad LOS and partially NLOS state, we can conclude that the combination of low SNR and poor channel state makes the effect of the Doppler effect small, since the bad large-scale characteristics dominate system performance. As the SNR increases, the channel performance improves and the small-scale differences become prominent. These high SNR variations are due to the appearance of the Doppler effect [70]. Vehicles in the fifth scenario (urban-NLOSv) move slower than the second scenario (highway-NLOSv), causing less fading and improving the performance by a tiny factor.

SOVA has the worst overall performance, even though it behaves slightly better than polar and LDPC decoding for low SNR. Although LDPC decoding starts from an error level similar to BCJR, initially, it has slow BER convergence, which gradually accelerates as the SNR increases. The main characteristic of LDPC is the presence of error floor, which is sometimes smoother and sometimes steeper, depending on the scenario. BER in polar decoding starts from a significantly larger initial BER, which is bigger than the uncoded case [71], but it rapidly decreases. The diminishing rate gradually increases until BER becomes zero. Polar is the only algorithm that has no error floor and the only one that its BER nulls for all the aforementioned scenarios.

From scenarios 1 to 5, we can deduce that BCJR, logBCJR and max logBCJR decoding algorithms have superior performance for low and middle SNR. Above this SNR, polar coding has better performance and is more suitable for coding implementations. Obviously, this is due to the fact that the parity bits of turbo codes contain more information for the data bits than the parity bits of LDPC, since the former are created by a convolutional process while the latter by a block process. Further, polar coding does not perform as efficiently as turbo because the frame binary data are too small to effectively apply the polarization effect, especially in poor SNR transmissions. Eventually, polar coding becomes superior at the high SNR region, because the BER declines rapidly in polar coding, while BER in turbo-based algorithms is limited by the presence of the error floor.

### 4.2. Simulation Results for 256-Bit Frame Length

**Figure 3:**
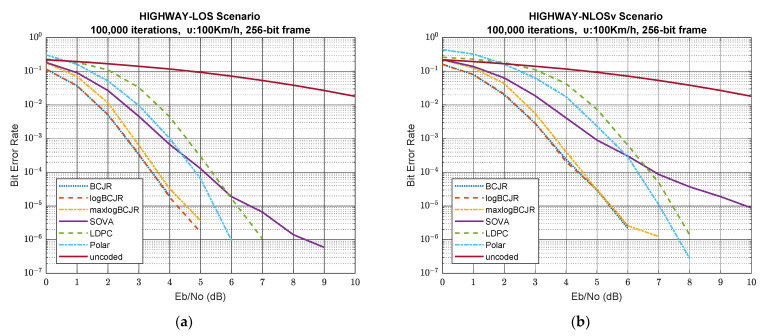

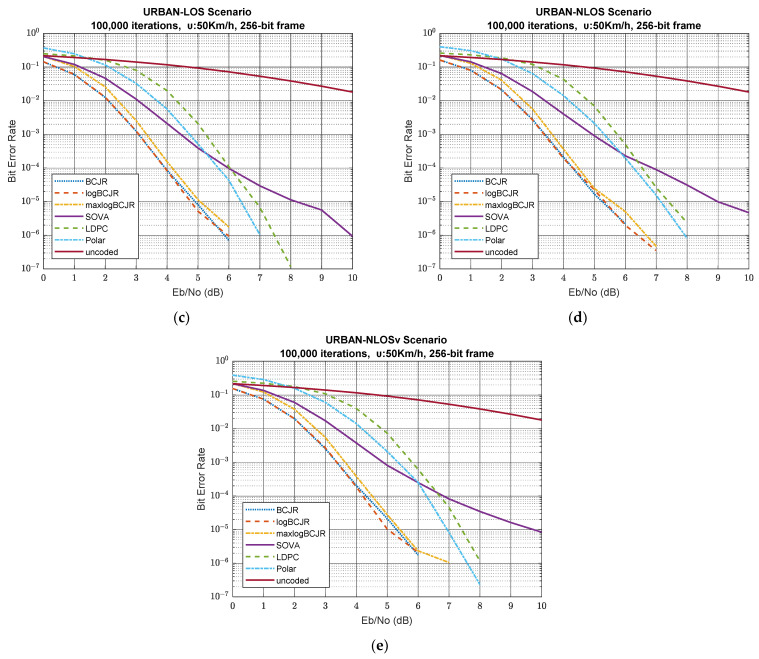
BER vs. SNR for scenarios 6-10 and 256-bit frame length. (**a**) highway-LOS scenario; (**b**) highway-NLOSv scenario; (**c**) urban-LOS scenario; (**d**) urban-NLOS scenario; (**e**) urban-NLOSv scenario.

Figure 3 shows BER performance of the simulated system for 256-bit frames, i.e., for scenarios 6 to 10 in Table 6. BCJR and logBCJR have almost identical performance and maxlogBCJR has a constant offset of 0.2 to 0.3 dB, driven by its suboptimal approximation in all five scenarios. Unlike the first five scenarios, the error floor is so smooth that it never appears and, eventually, BER in all three algorithms falls to zero in all 256-bit frame scenarios. Furthermore, the BER convergence rate is the same in these three algorithms and is increasing vastly. This leads to the BER becoming zero very fast, even in the NLOS and NLOSv scenarios. SOVA decreases more slowly as maxlogBCJR decreases, although it initially starts from the same BER. As a result, SOVA remains the worst turbo algorithm, independent of the traffic and channel state. Further, SOVA is worse than LDPC and polar coding in the high SNR region, although it has better performance in the low SNR region. Similar to the first five scenarios, SOVA does not zero BER below 10 db SNR, except in the case of the highway-LOS scenario. Even though polar coding does not have the best performance, its behaviour is the most interesting of all algorithms. It initially has the worst BER, even worse than the uncoded case, having an almost 40% error rate. Gradually, the rate of convergence increases and, eventually, the slope of BER is the steepest. LDPC has an inferior performance at the low SNR region, but above 2 to 3 dB, it starts to converge vastly at a pace similar to polar decoding. The error floor is absent from the LDPC in the 256-bit frame scenarios, except from the urban-NLOS scenario, in which it is negligible. Eventually, BER in all scenarios becomes zero. Unfortunately, LDPC and polar never attain the performance of BCJR-based algorithms because the latter converges rapidly at low SNR. To conclude, BCJR-based algorithms perform best, not only at low and middle SNR but also at high SNR for 256-bit frames in simulated scenarios 6–10.

Table 9 shows the SNR required to attain 0.001 and 0.000001 BER for all 256-bit frame scenarios. We can easily conclude that BCJR-based algorithms are the best choice in both cases. Considering both efficiency and complexity, maxlogBCJR is obviously the best candidate for 256-bit frames, as it is by far the least complex algorithm among them, and the gap from optimality is only 0.2 to 0.3 dB for the first case and 1 dB maximum for the second. Polar and LDPC fail to converge fast, resulting in having 1.7 and 2.2 dB offset, on average, for the first case, although their performance gradually accelerates as the SNR increases. SOVA is the only algorithm that reaches the second threshold only in the LOS-based scenarios.

Table 10 shows the BER efficiency for specific signal-to-noise ratios. From this table, we can extract many useful results, concerning the impact of the traffic model and the channel state on the system performance. From scenarios 6 and 7, we can conclude that an LOS channel state improves system performance for highway traffic around 10-times, on average, at low SNR, compared to the NLOSv channel state. Scenarios 8, 9 and 10 show that an LOS channel state increases the system’s performance for urban traffic above 10-times, on average, and approximately 40-times for BCJR-based algorithms at low SNR. Taking these facts into account, we can conclude that BCJR-based algorithms are more resistant in multi-obstacle environments, as is the case in urban areas. The high SNR case in Table 10 implies something more important. At 9 dB SNR, all algorithms drop to zero, excluding SOVA. This means that the choice of the algorithm is independent of the performance, so we can choose the less complex algorithm for such situations.

Scenarios 6 and 8 show the effect of the traffic model on the LOS channel state. The urban scenario needs a further 0.7 to 0.9 dB and less than 1 dB to achieve the 0.001 and 0.000001 BER objective, respectively. This means that the urban-LOS scenario needs approximately 1.25-times more power than the highway-LOS scenario to attain the same objectives.

The impact of different channel states on the urban traffic model is examined for scenarios 8, 9 and 10. From Table 9 and Table 10, we can conclude that an LOS channel is more affected by polar and BCJR-based algorithms at low SNR, in which BER is about 40-times bigger than the NLOS and NLOSv channel. At high SNR, the presence of the LOS channel has the same impact on all algorithms compared to the NLOS and NLOSv states. On the contrary, in the NLOS scenario, the urban traffic statistical features dominate and the channel statistical characteristics have a minor impact on system performance.

The effects of different traffic models on NLOSv channel state are highlighted in scenarios 7 and 10. As in scenarios 2 and 5, we conclude that they have minor differences due to common small-scale characteristics.

Scenarios 6 to 10 reveal the superiority of BCJR-based algorithms for 256-bit frames. BCJR-based algorithms further improved the BER performance because the convolutional coding process spreads information to all bits. As in the case of 128 bits, LDPC is suboptimal because the parity bits are not enough to provide the information needed to efficiently correct the data bits, especially in the low and medium SNR range, where noise significantly affects the received data. As the SNR increases, the inherent errors decrease and the ability of the LDPC to correct errors improves rapidly. Polar coding improved its performance, but not too much, because the frame size is too small to exploit the polarization.

### 4.3. Simulation Results for 512-bit Frame Length

**Figure 4:**
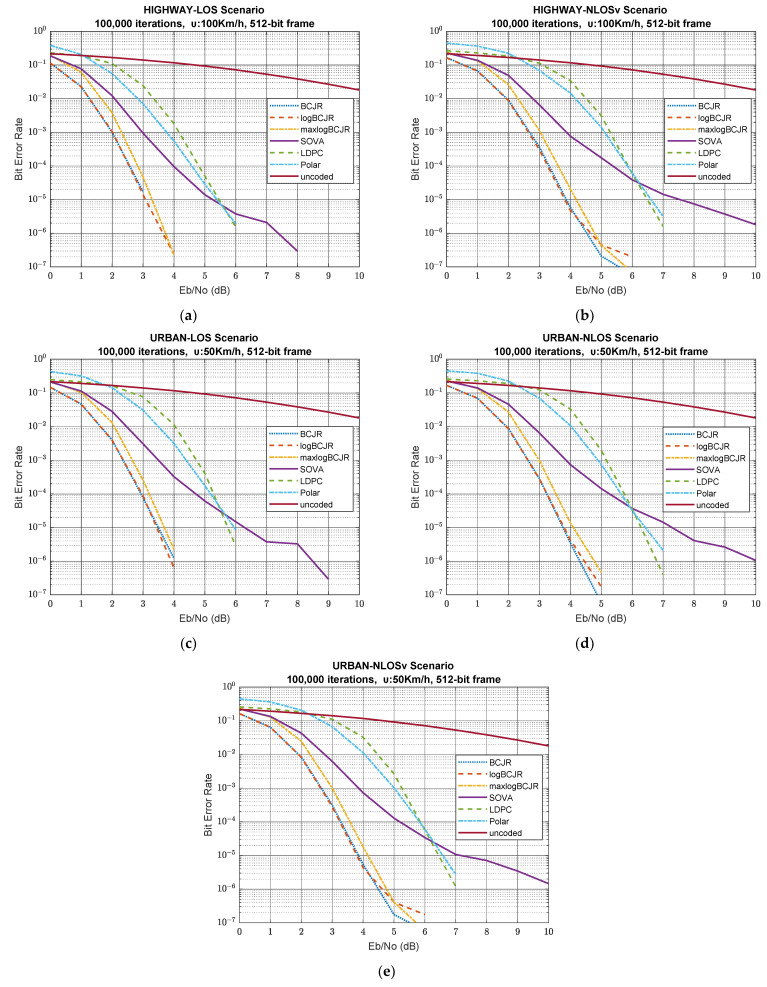
BER vs. SNR for scenarios 11-15 and 512-bit frame length. (**a**) highway-LOS scenario; (**b**) highway-NLOSv scenario; (**c**) urban-LOS scenario; (**d**) urban-NLOS scenario; (**e**) urban-NLOSv scenario.

Figure 4 presents BER efficiency of the simulated system for a 512-bit frame, i.e., for scenarios 11-15 in Table 6.

These scenarios are a clear example of the superiority of BCJR-based algorithms. BCJR and logBCJR converge fast initially, and above 2 dB SNR, the slope of convergence becomes very steep in all 512-bit scenarios. Apparently, maxlogBCJR maintains the same pace, but initially begins from a worse position. As a result, there is a gap from 0.2 to 0.3 that gradually decreases and, eventually, BER reaches the performance of the first two algorithms. BER in LDPC coding diminishes slowly at the beginning, and above 3 to 4 dB, it starts to converge at a high rate, which is similar to that of the BCJR. This sluggish startup is the reason for a constant gap of approximately 2.3 dB SNR between BCJR and LDPC above 3 dB. Although polar starts from the worst initial point, it starts to converge fast, but it lacks the pace of BCJR and LDPC. Initially, polar is worse than LDPC, but polar acquires better performance in the low SNR region, due to the slow BER reduction in LDPC. In the middle SNR region, polar has better performance, but LDPC has a higher rate of reduction and eventually becomes better at high SNR in scenarios 11–15. SOVA is the worst of all turbo algorithms but is better than LDPC and polar under 5.5 to 6.3 dB. Above this threshold, SOVA becomes the worst algorithm.

Table 11 summarises the SNR requirements that we need to achieve 0.001 and 0.000001 BER for scenarios 11, 12, 13, 14 and 15 and for 512-bit frame length. From this table, it is more than obvious that BCJR-based algorithms are dominant and have about 2 dB difference from the 5G-NR decoding algorithms.

Table 12 shows the performance for the decoding algorithms at 4 dB and at 9 dB SNR. From this table, we conclude that BCJR-based algorithms are the best choices for low-SNR cases. At high SNR, all algorithms have zero BER, except SOVA, so in this case, our best choice can be modelled by other parameters, such as complexity, latency and other QoS parameters, depending on the nature of the communication and the services that must be provided.

From Table 11 and Table 12, we can make performance comparisons related to the traffic model and the channel state for the case of 512-bit frame. The results, as expected, are similar to the 128- and 256-bit frame scenarios.

The impact of the channel state on the system performance is examined between scenarios 11 and 12 for the highway traffic model and among scenarios 13, 14 and 15 for the urban traffic model. Both groups of scenarios indicate that LOS channel state improves the decoding performance, but in a different way, which depends on the employed decoding algorithm and the traffic model. For example, LOS in LDPC has a great impact on the highway model, but this impact reduces in the urban model. Especially, at low SNR, there is little difference between LOS, NLOS and NLOSv, mainly because LDPC fails to converge fast in this SNR region. The BER performance of BCJR-based algorithms has one order of magnitude difference between LOS and NLOSv-NLOS states. Highway-LOS scenarios seem to have a greater effect than urban-LOS scenarios. The first category is two orders of magnitude better than the second one, but the latter is only one order of magnitude better.

From scenarios 11 and 13, we can deduce that LOS is affected by the different traffic models by an order of magnitude, on average, since the highway model is more reliable, having less obstacles than the urban model.

Scenarios 14 and 15 show that the impact of the urban model is dominant in comparison to the effect that the NLOS and NLOSv channel states produce, since their differences are negligible. This is a fact also in scenarios 12 and 15, where we can examine the effect of the traffic environment on the NLOSv channel state. These scenarios have almost identical behaviour, but the highway scenario is slightly worse due to bigger small-scale variations that have their origin in Doppler effect.

In summary, for scenarios 11 to 15, LDPC now has enough parity bits to exploit and become competitive in both the medium and high SNR ranges in order to achieve better BER performance than polar coding. Polar coding cannot increase its efficiency to the same extent as LDPC because the frame size remains too small to create good polarization conditions. BCJR-based algorithms further improve BER performance by taking advantage of the increased number of parity bits, which provide even more extrinsic information for the decoding process.

### 4.4. BER Performance Analysis for maxlogBCJR

In the previous subsections, we presented the simulation results for 128-, 256- and 512-bit frames, where miscellaneous results were observed. Other aspects that we can examine include the impact of the frame length, the velocity, the channel state and the traffic model on BER performance. The simulations show clearly that the research findings are common for all the decoding algorithms on average. For that reason, we choose maxlogBCJR as a representative algorithm and we will demonstrate its results only. For this purpose, we study the percent average improvement in BER based on these parameters.

**Figure 5:**
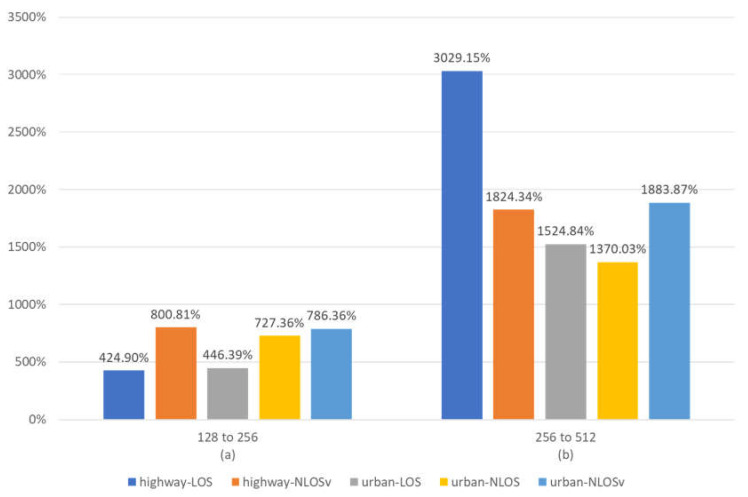
Mean percentage BER improvement vs. frame length for maxlogBCJR and for all simulated scenarios. (**a**) BER improvement when the frame length changes from 128 to 256 bits. (**b**) BER improvement when the frame length changes from 256 to 512 bits.

Figure 5 presents the impact of changing the frame length on all the simulation scenarios, i.e., highway-LOS, highway-NLOSv, urban-LOS, urban-NLOS and urban-NLOSv. Comparing Figure 5a with Figure 5b, we can observe the great impact that the frame length has on the BER performance. Although the frame size is doubled in both cases, in the second case, the improvement is much greater, indicating an exponential growth in efficiency, because the frame sizes are larger. The coding schemes for 128-bit frames are not efficient, due to the small number of parity bits. If we combine this with the poor performance that the NLOS and NLOSv scenarios have in general relative to the LOS, we can conclude that the LOS scenarios have greater BER performance from the NLOS and NLOSv scenarios. The coding schemes for 256 bits have more parity bits, accelerating the performance of the scenarios, but not in the same way. From Figure 5a, we can conclude that when the frame length changes from 128 to 256 bits, the NLOS and NLOSv scenarios have greater improvements compared to the LOS scenarios, mainly because the long error floors that affect the NLOS and NLOSv scenarios for 128-bit frames eventually vanish. Figure 5b shows that highway scenarios are more affected than urban scenarios when the frame size changes from 256 to 512 bits, because the good highway propagation model better exploits the increase in the parity bits.

**Figure 6:**
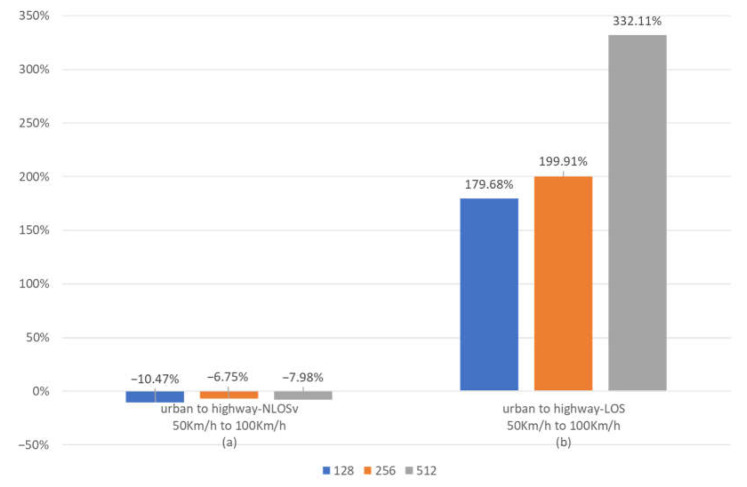
Mean percentage BER improvement when the velocity (traffic model) changes for maxlogBCJR and for 128-, 256- and 256-bit frames. (**a**) BER improvement for velocity change from 50 to 100 Km/h having NLOSv channel state. (**b**) BER improvement for velocity change from 50 to 100 Km/h having LOS channel state.

In Figure 6, we present the effect of change in speed, i.e., the change between the urban and highway traffic model, in both LOS and NLOSv channel states. To this end, the percent mean BER improvement versus the change in velocity is illustrated for 128-, 256- and 512-bit frames. Urban-NLOSv and highway-NLOSv scenarios (Figure 6a) have common large-scale characteristics, so the difference in performance is due to the doppler phenomenon only. From this figure, we can infer that by doubling velocity, the BER deteriorates by 6.75 to 10.47 percent. This comparison is the best representative of the study of the speed change, since in urban LOS and highway-LOS scenarios (Figure 6b), in addition to speed, different large-scale characteristics play an important role in the performance. The second comparison describes the impact of traffic model on BER efficiency for certain channel states, i.e., LOS. Given the small effect of the speed change, we conclude that the absence of obstacles in the highway-LOS scenario compared to the urban-LOS scenario results in a massive improvement in BER. This improvement is limited in the case of 128- and 256-bit frames due to the existence of an error floor, but the absence of error floor in 512-bit frames shows the substantial improvement brought about by the change of the traffic model.

**Figure 7:**
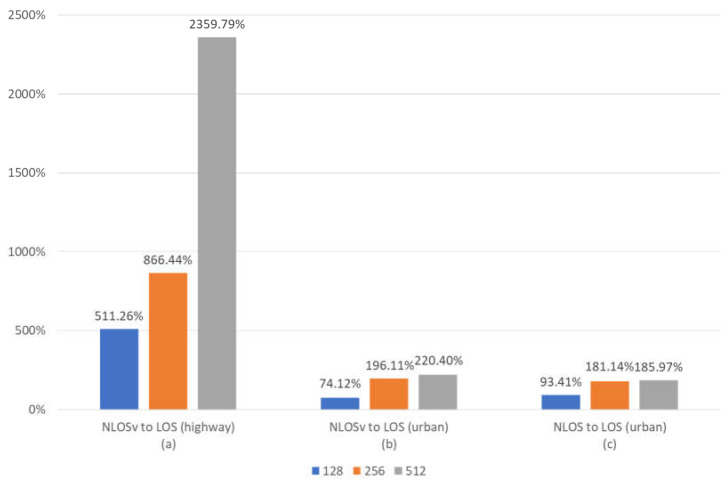
Mean percentage BER improvement when the channel state changes for maxlogBCJR and for 128-, 256- and 256-bit frames. (**a**) BER improvement for channel state change from to NLOSv to LOS in a highway traffic model. (**b**) BER improvement for channel state change from to NLOSv to LOS in an urban traffic model. (**c**) BER improvement for channel state change from to NLOS to LOS in an urban traffic model.

Figure 7 illustrates the impact of channel state on BER performance. We can conclude that the poor propagation environment in the NLOS and NLOSv cases dominates the performance, due to the permanent or partial absence of LOS component signals. This situation affects the system so severely that when the channel state changes to LOS, the difference in performance is significantly large in all cases (Figure 7a–c).

Of course, the impact of changing the channel state on BER efficiency is greater on the highway traffic model (Figure 7a), due to the presence of a strong LOS component. As a result, BER in the highway model is 4- to 10-times better than in the urban traffic model. Figure 7b,c show that NLOS and NLOSv have similar BER behaviour, since BER improves similarly, on average, when the channel state changes to LOS in urban areas. Another point to note is that the mean BER improvement is rapid when the channel state changes from NLOSv to LOS in the highway case (Figure 7a), and this acceleration largely depends on the frame length. Contrariwise, in the urban case (Figure 7b,c), the improvement is large but does not depend greatly on the frame length, due to the existence of the error floor. This error floor is more intense in the urban than in the traffic model affecting the obstacles that affect the communication quality and the BER performance more.

### 4.5. FER Simulation Results

**Figure 8:**
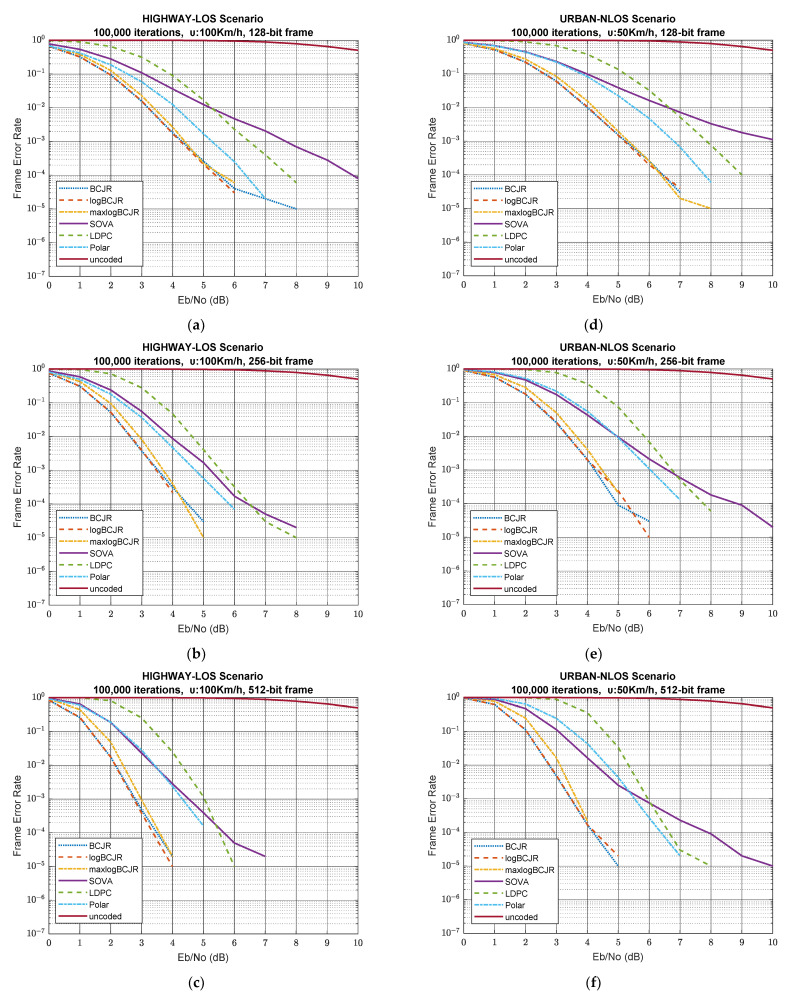
FER vs. SNR. (**a**) highway-LOS scenario 128-bit frames; (**b**) highway-LOS scenario and 256-bit frames; (**c**) highway-LOS scenario and 512-bit frames; (**d**) urban-NLOS scenario and 128-bit frames; (**e**) urban-NLOS scenario and 256-bit frames; (**f**) urban-NLOS scenario and 512-bit frames.

Prior to this, we presented and analysed the BER versus SNR simulation results. Another important parameter that must studied is FER versus SNR. The simulations show that the results have qualitative similarities for all the decoding algorithms on average. For that reason, we choose to show the FER performance for highway-LOS scenario (Figure 8a–c) and urban-NLOS scenario (Figure 8d–f) as representatives for the good and poor FER scenarios, respectively.

The results clearly show the superiority of turbo codes in both good and poor communication systems. In fact, as the data frame size increases, the difference in FER performance from polar and LDPC coding accelerates. The turbo implementations maintain the same correlations between them as in the BER results, since the BCJR and logBCJR performances are identical and the maxlogBCJR maintains an average gap of 0.2 to 0.3 dB. Further, SOVA coding remains, as expected, the least efficient, maintaining a slow rate of convergence across the entire SNR range. From Figure 8, we conclude that turbo coding is the best coding scheme based on FER performance for all scenarios and frame lengths.

The big qualitative difference between the BER and FER results is that FER is clearly better in polar coding than in LDPC in all simulated scenarios and for all frame sizes. In the case of the FER performance, this superiority extends over the entire SNR range, while, as we highlighted, the BER performance is worse in the low SNR region for all frame sizes and in the high SNR region for 512-bit frame sizes for polar coding. Of course, for the case of 512-bit frames, LDPC has a much higher convergence speed than polar coding, but it does not manage to reach its FER performance because its middle SNR region is characterized by a relatively slow rate of convergence. The difference in polar coding is also evident from the fact that its FER performance is consistently better than that of SOVA in the case of the highway-LOS scenario. This phenomenon can only be explained based on the distribution of binary errors in the data frames after the decoding process. Apparently, after the decoding process in LDPC, the binary errors are more uniformly distributed in the transmitted frames, leading to a large FER, while in polar coding, the errors are concentrated in specific frames. Since the initial errors before decoding are distributed in the same way for each coding scheme, we conclude that polar coding is much better at handling frames with few errors than LDPC and eliminating binary errors completely, producing error-free frames. On the contrary, it is less efficient for frames with a large number of errors, correcting only very few of them. LDPC is not as efficient as polar coding on frames with a small number of errors and, thus, does not produce many error-free frames. On the contrary, it is more efficient in cases of frames with many errors.

### 4.6. Comparison of the Proposed Approach with Published Literature

In Table 13, we compare the proposed approach presented in this paper with the recent published literature (since 2019). From the comparison presented in Table 13, only our proposed approach investigates 4G-LTE and 5G-NR coding performances for all V2X channel scenarios (highway-LOS, highway-NLOSv, urban-LOS, urban-NLOS, urban-NLOSv), as defined by 5G-V2X release 16 standard and for short V2X-compatible frame lengths.

## 5. Conclusions and Future Work

V2X systems have been very popular in the research community in the last few years. Ιt would be desirable to build a dynamic changing 5G V2X system with emphasis on channel coding and QoS. This article considers the three channel coding schemes used in LTE-V2X and 5G-NR V2X communication systems, i.e., turbo, polar and LDPC coding. Subsequently, a realistic, but with considerably reduced complexity, V2X system modelling methodology is proposed considering different propagation models. The novelty of this work is the combined approach and analysis of these propagation models and the different channel coding schemes. Finally, the incorporated stochastic model is analysed based on the recent 5G-V2X standard.

We investigate five different propagation models, i.e., highway-LOS, highway-NLOSv, urban-LOS, urban-NLOS and urban-NLOSv, for three different data frame lengths, i.e., 128-, 256- and 512-bit frames. Therefore, in total, we examined 15 simulation scenarios. Our analysis shows the dominance of turbo codes in small-length data-frame cases. Turbo codes proved to have superior performance in low and middle SNR regions, which are better than polar coding by approximately 1.5 dB in the LOS cases and 2 dB in NLOS and NLOSv cases. This gap is larger in LDPC, where turbo codes are superior by approximately 2 dB in LOS and 2.5 dB in NLOS and NLOSv cases. This difference seems to be typically independent of the frame length and the traffic model and depends only on the channel state condition. Of course, this phenomenon starts to vary at high SNR, where error floor occurs. These error floors are present only in turbo and LDPC coding and are absent from polar coding [72]. The performance of turbo codes is affected mainly in the 128-bit scenarios by the presence of error floors. These scenarios show that polar coding acquires superior performance above 7 dB in scenarios 1 and 3, i.e., LOS cases, above 8 dB in scenarios 2 and 5, i.e., NLOSv cases, and above 9 dB in scenario 4, namely the NLOS case. The simulation results for the 256-bit frame show that polar and LDPC diminish the gap at 1 to 1.5 dB, but they never reach the performance of turbo coding, as BER in the latter zeroes rapidly. Finally, the 512-bit frame simulation results show that turbo codes retain their superiority, unchanged at the high SNR region. Polar coding has a very a fast rate of convergence, especially in 128- and 256-bit scenarios, but it has a severe drawback. Polar coding in all simulations has BER equal to 0.4 at 0 dB. This rate is 0.1 dB far from been totally random, i.e., having the same efficiency as flipping a coin. As a result, polar coding has a long way to go to reach the performance of the BCJR-based algorithms and never actually reaches it. On the other hand, LDPC converges very slowly at low SNR and, although it has a great pace of convergence afterwards, cannot maintain the performance of BCJR-based algorithms. To conclude, we showed that turbo codes are the best choice for small data frame lengths in a 5G-V2X communication environment, excluding the case of very small data frames (128 bits) and high SNR cases, where polar coding has better BER performance.

A Vehicular Ad Hoc Network (VANET) is a big step towards intelligent transportation systems. Therefore, many manufacturers are supplying vehicles with onboard computing and communication devices, in-car sensors and navigation systems for the deployment of large-scale vehicular networks. By using different sensors, vehicles can collect and interpret information with very high reliability and minimum delay, with the purpose of helping the driver to make a decision, particularly in driver assistance systems. Specifically, applications where vehicles move in coordination, such as a platoon of vehicles, require secure wireless communication to ensure reliable connectivity and safety [73]. These requirements depend strongly on the design of the communication model, especially the physical layer. Therefore, historical data will be needed for the effectiveness of V2X communication models, e.g., different channel coding schemes, under realistic propagation models. These data can be used by industry to train dynamic systems that will select the appropriate parameters of the V2X communication model, depending on the conditions of the physical environment.

This study is a springboard to extensive research for the utilisation of turbo coding in small-frame 5G-V2X communications, in terms of communication aspects, in addition to BER performance. Thus, as future work, the impact of other parameters, such as reliability, end–end latency, data rate per vehicle and communication range, should be examined. Further, the behaviour of BER performance in a dynamic communication system, where the communication model and channel state vary in time according to some probabilistic criteria, must be examined. Furthermore, larger frame lengths can be investigated. In that way, we can examine if turbo coding retains its superiority in a more realistic vehicle scenario, where channel states change between LOS, NLOS and NLOSv, and traffic models change between urban and highway in the same communication scenario in real time. Another point of interest is the comparison of our model with geometry-based stochastic models and the confirmation that the latter produce the same qualitative results as presented in this article. Moreover, we can create a channel model that incorporates both Reconfigurable Intelligent Surfaces [74,75,76], as beyond-5G (B5G) and -6G infrastructure candidates, as well as beamforming [77,78,79].

Finally, another aspect to be considered in the future is extensive investigation of a holistic and dynamically reconfigurable coding scheme that uses BER performance and QoS attributes for choosing the most suitable decoder for a specific SNR. Such a design could be supported by artificial intelligence principles and trade-offs should be determined among performance and computational complexity.

## Figures and Tables

**Table 1:** Path loss in dB for V2X system model.

Channel State	Traffic Model	Path Loss (dB)
LOS, NLOSv	urban	$PL_{LOS,u}\left(d,f_{c}\right)=38.77+16.7\log_{10}d+18.2\log_{10}f_{c}$
	highway	$PL_{LOS,h}\left(d,f_{c}\right)=32.4+20\log_{10}d+20\log_{10}f_{c}$
NLOS	urban	$PL_{NLOS,u}\left(d,f_{c}\right)=36.85+30\log_{10}d+18.9\log_{10}f_{c}$

**Table 2:** Shadow fading model parameters for V2X system model.

Channel State	Traffic Model	Path Loss (dB)	
		$\mathrm{Mean}\;\mu_{sf}$	$\mathrm{Std}.\;\mathrm{Deviation}\;\sigma_{sf}$
LOS, NLOSv	urban	$\mu_{sf}=0$	$\sigma_{sf}=3\;\mathrm{dB}$
	highway	$\mu_{sf}=0$	$\sigma_{sf}=3\;\mathrm{dB}$
NLOS	urban	$\mu_{sf}=0$	$\sigma_{sf}=4\;\mathrm{dB}$

**Table 3:** NLOSv additional blockage loss.

NLOSv Cases	NLOSv Additional Blockage Loss $max\left(0,\alpha \right),\;\;\alpha \sim Lognormal\left(\mu_{\alpha },\sigma_{\alpha }^{2}\right)$	
	$\mathrm{Mean}\;\mu_{a}$	$\mathrm{Std}.\;\mathrm{Deviation}\;\sigma_{\alpha }$
$max\left\{h_{T},h_{R}\right\}<h_{B}$	$\mu_{a}=9+max\left(0,15log_{10}d-41\right)$	$\sigma_{a}=4.5$
$min\left\{h_{T},h_{R}\right\}>h_{B}$	$\mu_{a}=0$	$\sigma_{a}=0$
else	$\mu_{a}=5+max\left(0,15log_{10}d-41\right)$	$\sigma_{a}=4$

**Table 4:** Large-scale fading in V2X communication models.

Channel State	Traffic Model	Path Loss (dB) + Shadow Fading + Blockage Loss	Random Loss Terms
LOS, NLOSv	urban	$PL_{LOS,u}\left(d,f_{c}\right)=38.77+16.7\log_{10}d+18.2\log_{10}f_{c}+\chi_{sf}+max\left(0,\alpha \right)\;$	$\chi_{sf}∼Lognormal\left(0,9\right)$ and $\alpha ∼Lognormal\left(\mu_{\alpha },\sigma_{\alpha }^{2}\right)$, according to Table 3
	highway	$PL_{LOS,h}\left(d,f_{c}\right)=32.4+20\log_{10}d+20\log_{10}f_{c}\;$ $+\chi_{sf}+max\left(0,\alpha \right)\;$	
NLOS	urban	$PL_{NLOS,u}\left(d,f_{c}\right)=36.85+30\log_{10}d+18.9\log_{10}f_{c}\;$ $+\chi_{sf}$	$\chi_{sf}∼Lognormal\left(0,16\right)$

**Table 5:** K-factor distribution parameters for LOS, NLOSv and NLOS states [7,8].

Channel State	Traffic Model	Κ Factor (dB)	
		$\mathrm{Mean}\;\mu_{K}$	$\mathrm{Std}.\;\mathrm{Deviation}\;\sigma_{K}$
LOS	urban	3.48	2
	highway	9	3.5
NLOSv	urban, highway	0	4.5
NLOS	urban	Rayleigh	

**Table 6:** Simulation scenarios.

Simulation Scenario	Data Frame Size	Traffic Model	Channel State
1	128	Highway	LOS
2	128	Highway	NLOSv
3	128	Urban	LOS
4	128	Urban	NLOS
5	128	Urban	NLOSv
6	256	Highway	LOS
7	256	Highway	NLOSv
8	256	Urban	LOS
9	256	Urban	NLOS
10	256	Urban	NLOSv
11	512	Highway	LOS
12	512	Highway	NLOSv
13	512	Urban	LOS
14	512	Urban	NLOS
15	512	Urban	NLOSv

**Table 7:** SNR in dBs for BER values of 10^−3^, 10^−6^ and 128-bit frame length.

BER	Scenario	BCJR	logBCJR	maxlogBCJR	SOVA	LDPC	Polar
10^−3^	1	3.4	3.4	3.6	4.9	5.2	4.7
	2	4.4	4.4	4.7	6.8	6.8	6.3
	3	3.8	3.8	4.1	5.9	6	5.6
	4	4.3	4.3	4.5	6.8	6.7	6.2
	5	4.3	4.3	4.5	6.6	6.7	6.2
10^−6^	1	6.8	6.8	7.2	-	>7	>8
	2	-	-	-	-	-	>8
	3	8.4	8.2	7.8	-	10	>7
	4	9.6	9.6	9.6	-	-	>9
	5	10	-	10	-	-	>8

**Table 8:** BER values for 4 and 9 dB SNR, 128-bit frame.

SNR (dB)	Scenario	BCJR	logBCJR	maxlogBCJR	SOVA	LDPC	Polar
4	1	2.1 × 10^−4^	2.3 × 10^−4^	3.4 × 10^−4^	3.5 × 10^−3^	1.1 × 10^−2^	3.5 × 10^−3^
	2	2 × 10^−3^	1.8 × 10^−3^	2.6 × 10^−3^	1.2 × 10^−2^	5 × 10^−2^	2.6 × 10^−2^
	3	8 × 10^−4^	8 × 10^−4^	10^−3^	8 × 10^−3^	3 × 10^−2^	1.5 × 10^−2^
	4	2 × 10^−3^	2 × 10^−3^	2.5 × 10^−3^	10^−2^	6 × 10^−2^	3 × 10^−2^
	5	2 × 10^−3^	2 × 10^−3^	2.5 × 10^−3^	1.2 × 10^−2^	5 × 10^−2^	3 × 10^−2^
9	1	2.3 × 10^−7^	2.3 × 10^−7^	3.5 × 10^−7^	4.5 × 10^−5^	0	0
	2	1.4 × 10^−6^	2.1 × 10^−6^	3.2 × 10^−6^	2.2 × 10^−4^	2.3 × 10^−5^	0
	3	4 × 10^−7^	3 × 10^−7^	4 × 10^−7^	1.5 × 10^−4^	5 × 10^−6^	0
	4	2 × 10^−6^	3 × 10^−6^	3 × 10^−6^	2.5 × 10^−4^	6 × 10^−6^	1.5 × 10^−6^
	5	1.5 × 10^−6^	2 × 10^−6^	3 × 10^−6^	2 × 10^−4^	1.5 × 10^−5^	0

**Table 9:** SNR in dBs for BER values of 10^−3^ and 10^−6^, 256-bit frame.

BER	Scenario	BCJR	logBCJR	maxlogBCJR	SOVA	LDPC	Polar
10^−3^	6	2.7	2.7	2.9	3.8	4.6	4.0
	7	3.5	3.5	3.7	4.9	5.8	5.4
	8	3.2	3.2	3.5	4.4	5.3	4.7
	9	3.5	3.5	3.8	4.9	5.7	5.3
	10	3.4	3.4	3.6	4.9	5.8	5.4
10^−6^	6	>5	>6	>6	8.4	>7	>6
	7	>6	>6	>7	-	>8	7.6
	8	6	5.8	>6	10	7.5	>7
	9	>6	6.4	6.7	-	>8	>8
	10	>6	>6	>7	-	>8	7.6

**Table 10:** BER values for 4 and 9 dB SNR, 256-bit frame.

SNR (dB)	Scenario	BCJR	logBCJR	maxlogBCJR	SOVA	LDPC	Polar
4	6	1.9 × 10^−5^	1.8 × 10^−5^	3.2 × 10^−5^	6.7 × 10^−4^	4.5 × 10^−3^	10^−3^
	7	2.4 × 10^−4^	2.1 × 10^−4^	4.1 × 10^−4^	4.1 × 10^−3^	4.4 × 10^−2^	1.7 × 10^−2^
	8	8.5 × 10^−5^	8.2 × 10^−5^	1.5 × 10^−4^	2.1 × 10^−3^	1.9 × 10^−2^	5.6 × 10^−3^
	9	2.1 × 10^−4^	1.9 × 10^−4^	3.6 × 10^−4^	4.1 × 10^−3^	4.3 × 10^−2^	1.4 × 10^−2^
	10	2.1 × 10^−4^	1.9 × 10^−4^	3.9 × 10^−4^	3.8 × 10^−2^	4 × 10^−2^	1.4 × 10^−2^
9	6	0	0	0	5.8 × 10^−7^	0	0
	7	0	0	0	1.9 × 10^−5^	0	0
	8	0	0	0	5.6 × 10^−6^	0	0
	9	0	0	0	9.8 × 10^−6^	0	0
	10	0	0	0	1.7 × 10^−5^	0	0

**Table 11:** SNR in dBs for BER values of 10^−3^ and 10^−6^, 512-bit frame.

BER	Scenario	BCJR	logBCJR	maxlogBCJR	SOVA	LDPC	Polar
10^−3^	11	2	2	2.3	3	4.2	3.7
	12	2.7	2.7	3	3.9	4.3	4.1
	13	2.4	2.4	2.7	3.5	4.7	4.4
	14	2.7	2.7	3	3.9	4.2	3.9
	15	2.7	2.7	3	3.9	4.3	4
10^−6^	11	>3	3.7	3.8	7.4	>6	5.8
	12	4.6	4.7	4.8	-	>7	>7
	13	>4	3.9	>4	8.5	>6	>6
	14	4.4	4.5	4.8	10	6.8	>7
	15	4.6	4.5	4.8	-	>7	>7

**Table 12:** BER values for 4 and 9 dB SNR, 512-bit frame.

SNR (dB)	Scenario	BCJR	logBCJR	maxlogBCJR	SOVA	LDPC	Polar
4	11	0	2.3 × 10^−7^	2.3 × 10^−7^	9.7 × 10^−5^	1.8 × 10^−3^	5.5 × 10^−4^
	12	5.9 × 10^−6^	4.8 × 10^−6^	2.1 × 10^−5^	7.7 × 10^−4^	3.4 × 10^−2^	1.5 × 10^−2^
	13	1.2 × 10^−6^	6.4 × 10^−7^	2.3 × 10^−6^	3.2 × 10^−4^	1.1 × 10^−2^	3.2 × 10^−3^
	14	3.5 × 10^−6^	4.3 × 10^−6^	1.4 × 10^−5^	7.4 × 10^−4^	3.3 × 10^−2^	1.1 × 10^−2^
	15	5.4 × 10^−6^	4.3 × 10^−6^	1.8 × 10^−5^	7.3 × 10^−4^	3.2 × 10^−2^	1.1 × 10^−2^
9	11	0	0	0	2.9 × 10^−7^	0	0
	12	0	0	0	3.7 × 10^−6^	0	0
	13	0	0	0	2.9 × 10^−7^	0	0
	14	0	0	0	2.6 × 10^−6^	0	0
	15	0	0	0	3.5 × 10^−6^	0	0

**Table 13:** Comparison of our proposed approach with published literature. The symbol ✓ defines that the field has been implemented and examined in the corresponding references.

Related References	Channel Coding						V2X rel. 16 Channel Consideration	Performance Parameter		Short Frame Length
	BCJR	Log BCJR	Maxlog BCJR	SOVA	LDPC	Polar		BER	FER	
[33]		✓						✓	✓	✓
[34]		✓	✓				✓	✓	✓	✓
[35]		✓	✓	✓			✓	✓		✓
[36]			✓		✓	✓			✓	
[37]			✓		✓			✓		
[38]			✓		✓			✓		
[39]			✓		✓	✓			✓	
[40]			✓		✓			✓		
[41]			✓		✓	✓		✓	✓	✓
[42]					✓	✓		✓		
Proposed approach	✓	✓	✓	✓	✓	✓	✓	✓	✓	✓

## Data Availability

Data is unavailable due to privacy or ethical restrictions.
